# Study of plants traditionally used in public and animal health management in Seharti Samre District, Southern Tigray, Ethiopia

**DOI:** 10.1186/s13002-015-0015-5

**Published:** 2015-03-15

**Authors:** Solomon Araya, Balcha Abera, Mirutse Giday

**Affiliations:** Department of Biology, Jimma University, Jimma, Ethiopia; Aklilu Lemma Institute of Pathobiology, Addis Ababa University, Addis Ababa, Ethiopia

**Keywords:** Medicinal plants, Preference ranking, Seharti Samre, Traditional medicine, Ethiopia

## Abstract

**Background:**

In Ethiopia, medicinal plants have continued to play vital role in fulfilling human and livestock healthcare needs of different communities. However, these valuable resources are being depleted mainly due to agricultural expansion and deforestation. Therefore, immediate action is required to conserve these resources and document the associated knowledge. The purpose of this study was, thus, to document and analyze information associated with medicinal plants that are used in managing public and animal health problems in Seharti Samre District, Southern Tigray, Ethiopia.

**Methods:**

Ethnobotanical data were collected from July 1, 2011 to December 30, 201 mainly using semi-structured interviews with informants sampled using purposive sampling technique and through field observations.

**Results:**

The study revealed the use of 90 medicinal plant species in Seharti Samre District for the treatment of several human and livestock diseases. The plants belonged to 46 families and 82 genera. The majority of the medicinal plants were indicated to be harvested from the wild. Leaf was the most frequently harvested plant part accounting for 44% of the reported plants, followed by roots (16%), whole plants (10%) and seeds (8%). The most widely used method of preparation was crushing (37%), pounding (15%) and chewing (13%). Most medicinal plants were applied internally (64.6%), followed by external application on the skin (35.4%). Febrile illness is the disease group in the study area that scored the highest ICF value (0.97), followed by cardio-vascular problems (0.97) and evil eye (0.95). Different preference ranking exercises were also used to determine the most preferred and potential medicinal plants in the study area.

**Conclusion:**

In Seharti Samre District, medicinal plants are still playing important role in the management of various human and livestock diseases, many of which are harvested for their leaf parts. However, activities of claimed medicinal plants need to be evaluated before recommending them for their wider use. Evaluation priority should be given to medicinal plants with the highest informant agreement as such plants are believed to have better activity.

## Background

The problem of health in African countries, including Ethiopia, is very acute as people have no full access to government and private health services. The absence or inaccessibility of modern healthcare services and other factors such as high cost of modern drugs and services and better curing of herbal remedies against some chronic diseases has caused a large percentage of the population to rely on traditional medicine, and mostly on herbal remedies [1,2], for its primary health-care needs. In Africa, up to 80% of the population relys on traditional medicine to help meet its health care needs [3].

Ethiopia is a land of high variation in landscape, flora and fauna, multiplicity of ethnic groups with complex multicultural diversity, languages, cultures and beliefs which have in turn contributed to the high diversity of traditional knowledge and practices of the people including the use of medicinal plants. In Ethiopia, medicinal plants play important role in fulfilling human and livestock health care needs of different communities. Traditional use of medicinal plants has remained as the main alternative solution for different human and livestock health problems largely due to shortage of pharmaceutical products and modern health service stations, unaffordable prices of conventional drugs and drug resistance [4].

Today, many Ethiopian medicinal plants are facing extinction or severe genetic erosion mainly due to agricultural expansion, deforestation, over exploitation and destructive harvesting. *Securidaca longipedenculata* and *Warburgia ugandensis* are among the popular medicinal plants in Ethiopia that are being threatened due to over exploitation and destructive harvesting. *Hagenia abysinica* is another medicinal plant that is being depleted as a result of over exploitation [4]. For most of the threatened and endangered medicinal plants, no conservation action has been taken, and there is no even a complete inventory of these plants. Much of the knowledge on the uses of medicinal plants in the country is still held only by traditional societies and is usually transmitted verbally [5]. Unless the plants are conserved and the associated ethnomedicinal knowledge documented, there is a danger that both the valuable medicinal plants and the knowledge could vanish forever. As it is happening elsewhere in the country, medicinal plants of the Seharti Samre District of Tigray are facing the danger of being lost unless appropriate documentation and conservation measures are taken.

A number of ethnobotanical studies have previously been conducted in different parts of Tigray to document the use of medicinal plants [6-14]. For example, studies conducted by Teklay et al. [12], Abdurhman [9] and Zenebe et al. [13] reported the use of 114, 113 and 68 medicinal plants in Kilte Awlaelo, Ofla and Asgede Tsimbila districts, respectively. However, there is no record that indicates the documentation of medicinal plants used by the people of Seharti Samre District. The purpose of this study was, therefore, to document and analyze traditional knowledge of medicinal plants used to manage human and animal health problems in Seharti Samre District, Southern Tigray, Ethiopia.

## Methods

### Description of the study area

**Figure 1:**
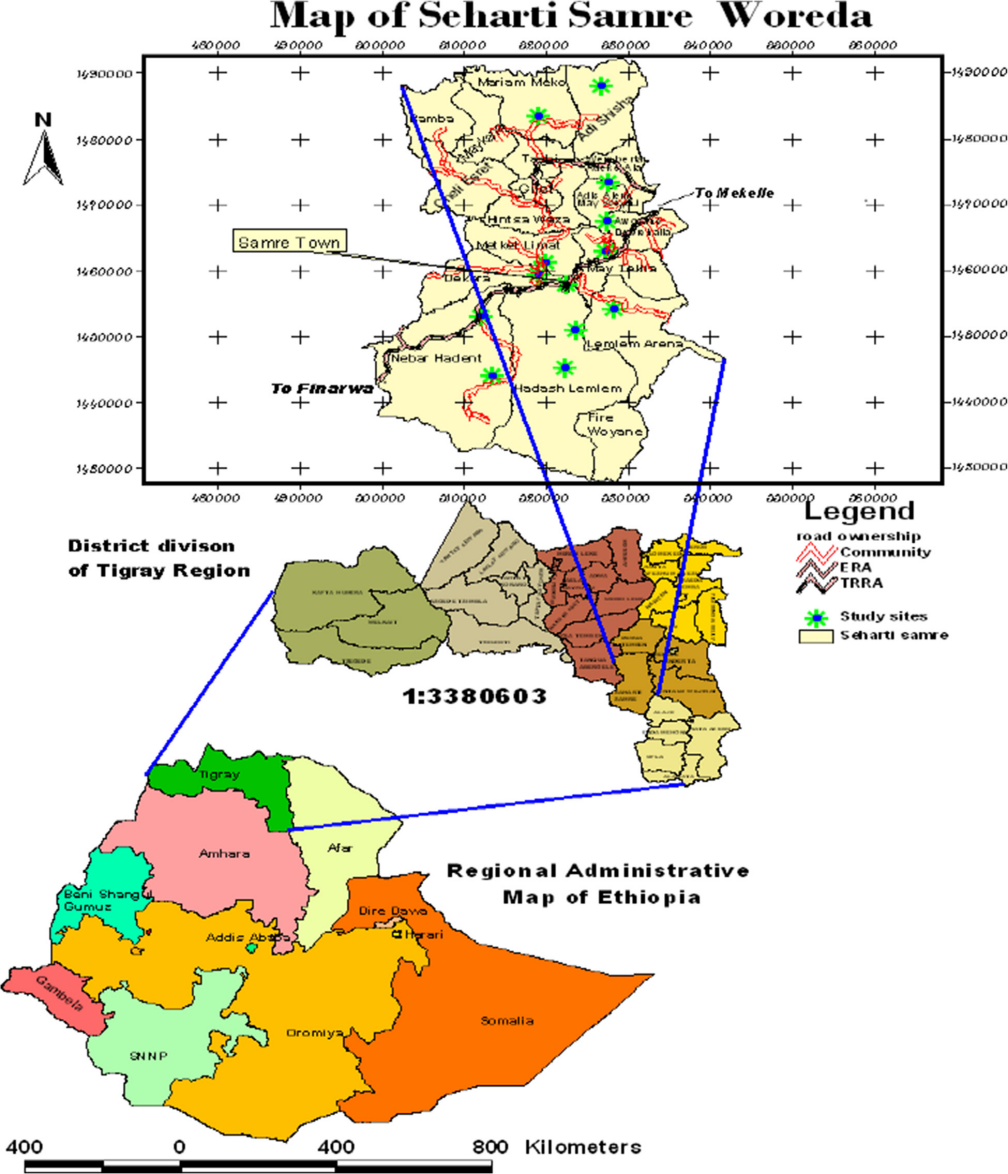
**Map of the study area (data source: Ethio GIS).**

The study was conducted in Seharti Samre District (*Woreda*) located between 12^0^30’ and 13^0^02’ latitude north and 38^0^59’ and 39^0^26’ longitude east in south east of Tigray at about 57 km southwest of Mekelle, the capital city of Tigray Region and 820 km north of Addis Ababa (Figure 1). The District has undulated type of landscape with altitude ranging from 1470 to 2370 meter above sea level (m.a.s.l) (Seharti Samre District Rural Agricultural Office, unpublished data of 2011). The District has warm and hot climate conditions and unimodal rainfall distribution that extends from April to September with the highest peak in July and August.

The great majority of inhabitants in the District belong to Tigray ethnic group. According to unpublished report of 2011 obtained from Seharti Samre District Health Office, the public healthcare coverage of the District reaches 85%. However, there are people who still rely on traditional medicine due to low cost of the service and more preference to the system. A study by Yirga [8] reported the use of 27 medicinal plants in the neighbouring District of Enderta. In the District, there are five health centres and eighteen health posts. Pneumonia, skin diseases, malaria, intestinal parasites infection and diarrhoea, acute respiratory tract infection, gastritis, urinary tract infection, diarrhoea, snake bites and conjunctivitis are the top ten human health problems in the District (Seharti Samre District Health Office, unpublished report, 2011). Anthrax, black-leg, trypanosomiasis, bloat, gland swelling, cough and intestinal diseases are the major livestock diseases in the District (Seharti District Samre Veterinary Health Office, unpublished report, 2011).

### Selection of study sites

The study was conducted in Seharti Samre District from July 1, 2011 to December 30, 2012. Prior to conducting this study, proposal approval letter was received from Jimma University Ethical Review Committee (ERC) and verbal informed consent from each informant who participated in the study.

### Sampling of informants

Purposive sampling method was employed to select 66 traditional healers and knowledgeable individuals (55 men and 11 women) between the ages of 20 and 76 years for semi-structured interviews. Informants that were involved in preference/priority and direct matrix rankings were selected randomly from those healers and knowledgeable individuals that were already sampled for the interviews.

### Collection of data and plant specimens

Semi-structured interviews and field observation [15,16] were employed to collect ethnobotanical data. Individual interviews were held with informants to gather data on medicinal plants with regards to plants parts used, methods of preparation, dosage, route of administration, diseases treated, threats, conservation status, cultivation practice, marketability, acquisition/transfer of indigenous knowledge and attitudes of people towards using such plants. All communications with informants were held in Tigrigna, the official language of Tigray Region. Specimens of medicinal plans were collected, dried and identified with the help of botanists at Aklilu Lemma Institute of Pathobiology and the National Herbarium, Addis Ababa University (AAU) and were deposited at the Jimma University Herbarium.

### Data analysis and presentation

Microsoft Excel spreadsheet software was employed for organizing and analysis of ethnobotanical data. Descriptive statistical analysis was employed to determine the number of medicinal plants used and ailments treated in the study District, the most frequently used plant parts, main routes of remedy administration and to identify popular medicinal plants, main ways of knowledge acquisition/transfer, major habitats of the plants and their marketability.

Informant consensus factor (ICF) was calculated for each ailment group to estimate level of agreement among informants in the selection of plants against a given category. ICF was calculated using the formula $\mathrm{I}\mathrm{C}\mathrm{F}=\frac{nur- nt}{nur-1}$ [17] where *ICF* stands for informant consensus factor, *nur* for number of use citations in each category and *nt* for number of species used.

Preference ranking technique [18] was used to identify the most preferred medicinal plants used in the District to treat snake bite based on informants’ personal preference or perception. Snake bite is one of the ten most important human health problems in the District. It is also among the ten diseases with the highest ICF values. The most preferred plant was assigned the highest score (6), while the least effective one was given the lowest value (1). For this purpose, eight individuals were randomly selected from the people that had already served as key informants. Each informant was provided with fresh specimens of six medicinal plants having the highest frequency of report by informants for being used to treat snake bite. The informants were then asked to rank the plants according to their degree of preference.

Priority ranking exercise [18] was also performed by seven informants to rank different factors perceived as threats to medicinal plants in the study area based on level of destructive impacts. During exercises, informants assigned values 1–4, 1 for the least destructive threat and 4 for the most destructive one.

Direct matrix ranking [16,18] was performed for six commonly reported multipurpose medicinal plants. Based on the relative benefits obtained from each plant, a group of five informants were asked to discuss and assign, to each attribute, a value between 1 and 4 (1 for the lowest value and 4 for the highest value). Scores were then added and plants ranked.

## Results

### Acquisition/transfer of medicinal plants knowledge

Majority (65.6%) of informants reported that transfer of knowledge on medicinal plants in the study District took place along the family line, from parents to children. Some informants (21.2%) reported close relatives as sources of knowledge while other informants (9.1%) indicated transfer of the knowledge on payment (9.1%) and few reported acquisition of knowledge through trial and error methods (6.1%). Results of interviews also revealed that 69.7% of the informants were willing to transfer their knowledge of medicinal plants along the family line verbally and 9.1% reported that they were happy to transfer the knowledge verbally assisted by practical demonstration. Other informants (21.2) reported that they had no interest to transfer their knowledge at all.

### Comparison of medicinal plant knowledge between age groups

Analysis was made to compare medicinal plant knowledge among two age groups Result revealed that members belonging to the age group above 40 year reported an average of 2 medicinal plants while those belonging to the age group between 20 and 40 years reported an average of less one medicinal plant. During interviews and field visits, informants above 40 years of age were found to be very conversant on how to collect plants, process remedies and administer them. Besides, older informants had stronger belief in the curative effect of their medicinal plants as compared to the younger generation.

### Medicinal plants reported and diseases treated

Ninety medicinal plant species that were used for the treatment of 51 human (Table 1) and 25 animal diseases (Table 2) were reported by the informants in Seharti Samre District. Of the total medicinal plants, 62 were used to treat human diseases only, 25 to treat both human and animal diseases and three to mange animal diseases only. The medicinal plants belonged to 46 families and 82 genera. The family Solanaceae was represented by 9 species, Lamiaceae by 8 species, Fabaceae by 6 species, Asteraceae and Euphorbiaceae by 5 species each, Malvaceae by 4 species, Boraginaceae and Capparidaceae by 3 species each. The families Rutaceae, Asclepiadaceae, Apocynaceae, Brassicaceae, Cucurbitaceae, Oleaceae, Rhamnaceae and Vitaceae contributed 2 species each and the remaining 28 families were represented by one species each. Most of the recorded medicinal plants were shrubs and herbs accounting for 42.2% and 39%, respectively, followed by trees (14.4%) and climbers (4.4%).

**Table 1:** **List of medicinal plants used to treat human diseases**

**Scientific name**	**Family**	**Local name**	**Habit**	**Parts used**	**Disease treated**	**Mode of preparation and administration**	**Application route**	**Voucher no**
*Achyranthes aspera* L.	Amaranthaceae	mechelo	Herb	Root	Arthritis	Roots chopped into pieces; seven pieces are put on clean thread and tied on the waist until recovered from the disease	Dermal	SA01303
				Leaf	Herpes zoster	Leaves roasted on metal plate, pounded into powder , mixed with pure butter and smeared on affected part	Dermal	
*Acokanthera schimperi* (A.DC.) Schweinf.	Apocynaceae	Mebtie (merez)	Tree	Leaf	Jaundice	Leaves are boiled in water for an hour and the patient takes a cup of the solution per day for seven days	oral	SA01333
					Wound	Leaves are crushed and paste smeared on affected part	Dermal	
*Allium sativum L.*	Alliaceae	Tsa’da shegurti	Herb	Bulb	Gastritis	Bulb is eaten with flatbread locally known as ‘enjera’	oral	SA01368
					Evil eye	Smelling aroma of bulb	Nasal	
					Wound	Bulbs are crushed, squeezed and wound washed with the liquid until healed	Dermal	
					Ring worm	Rubbing affected area with bulb	dermal	
					Malaria	Bulb of *Allium sativum*, *Artemisia afra*, *Ruta chalepensis* and *Lepidium sativum* are crushed together and paste taken for five-seven days	oral	
*Aloe megalacantha* Baker	Alloaceae	Ere	Shrub	Exudate	Malaria	Exudate, mixed with honey, is taken orally with coffee cup for three days	oral	SA01384
					Diabetes	Coffee cup of exudate taken every morning for long period of time	oral	
					Impotence	Smearing penis with exudate	Dermal	
					Dandruff	Exudate smeared on head skin for a week	Dermal	
					wound	Exudates smeared on wound		
*Alysicarpus ferrugineus* Hochst. & Steud. ex A. Rich.	Fabaceae	Hambo hambo bita	Herb	Root	Jaundice	Root chewed for five days	oral	SA01336
*Argemone mexicana* L.	Papaveraceae	Medafe tilian	Herb	Leaf	wound	Fresh leaves are collected, crushed and paste applied on affected part	Dermal	SA01381
					eczema	*Argemone mexicana* leaves crushed and powder sprayed on affected part. After two days, leaves of *Dodonaea angustifolia* roasted on iron sheet, pounded into powder are again sprayed on affected part	Dermal	
*Artemisia afra* Jacq. ex willd.	Asteraceae	Chena baria	Herb	Leaf	Evil eye	Aroma of the leaves help in expelling evil eye	nasal	SA01309
					Evil eye	Leaves of *Artemisia afar* and *Ruta chalepensis* and bulb of *Allium sativum* are crushed and aroma sniffed	nasal	
*Asparagus africanus* Lam.	Asparagaceae	Kasta ansti	Shrub	Root	impotence	Roots are pounded into powder, mixed with meat soup and vegetable and taken every evening for a month	oral	SA01340
					Evil eye	Root of *Carissa spinarum* and leaves of *Ruta Chalepensis, Artemisia afra, Cucumis ficifolius* and A*sparagus africanus* are crushed and fumigated indoor	nasal	
*Boscia salicifolia* Oliv.	Capparidaceae	Shesha	Shrub	Leaf	Ear infection	Leaves are crushed, squeezed and liquid filtered with clean cotton and three drops are applied on infected ear	ear	SA01329
*Cadaba rotundifolia* Forssk.	Capparidaceae	Mora	Shrub	Leaf	toothache	Chew leaves and hold paste on affected tooth	oral	SA01328
*Calotropis procera* (Ait.) Ait.	Asclepiadaceae	Ginda	Shrub	Flower	Kidney stone	Dry flower crushed into powder and mixed with dough of wheat and medicine prepared tablet form is baked on iron plate and three to four tablets are taken for long period of time	oral	SA01375
				Latex	Haemorrhoids	Latex is smeared on affected area	Dermal	
					Wart	Cover the first appearing wart with latex	Dermal	
					Scabies	smear whole affected area with latex	Dermal	
					wound	Dress wound with latex	Dermal	
				Root	Tuberculosis	Roots of C. *procera* is crushed into powder and mixed with pounded bark of *Croton macrostachyus* and leaves of *Ficus palmata* and sniffed	nasal	
*Calpurnia aurea* (Alt.) Benth.	Fabaceae	Hetsawets	Tree	Seed	Gonorrhoea, syphilis	Seeds are roasted on iron sheet, ground into powder, mixed with honey, prepared in the form of tablet and three tablets are taken every day for five days. As a side effect, it causes headache	oral	SA01345
					Amoebiasis	Seeds are roasted on iron sheet, ground into powder, mixed with honey, dissolved in cup of water and taken for three days	oral	
*Capparis tomentosa* Lam.	Capparidaceae	Andiel	Shrub	Root	Evil eye	Patient fumigates himself with smoke of burning root		SA01337
*Carissa spinarum* (Forssk.) Vahl.	Apocynaceae	Agam	Shrub	Root	Evil eye	Crushed root is fumigated on a clay plate to expel evil eye	Nasal	SA01316
				Leaf	Febrile illness	Leaves are crushed, squeezed and liquid taken with coffee	oral	
				Fruit	Wound	Fruits are crushed, dried, pounded into powder and sprayed on wound	skin	
*Chenopodium murale* L.	Chenopodiaceae	Hamedmado, hamlikebbo	Herb	Leaf	Tetanus	Leaves are crushed, mixed with butter, roasted on metal plate and smeared on affected area before covering it with cotton cloth. Application is repeated three to four times within a week	Dermal	SA01332
					Vitiligo	Leaves are crushed and pasted applied on affected area	Dermal	
*Citrus lemon* (L.) Burm. f.	Rutaceae	Lomin	Shrub	Fruit	Haemorrhoids	Fruits and leaves are pounded, mixed with butter and applied on affected area	anal	SA01369
					Blood pressure	Fruit juice is added into cup of water and drunk every morning	oral	
					cough	Fruit decoction with sugar added into it is taken orally	oral	
					Tetanus	Crushed fruit is mixed with butter and applied on affected area and is covered with clean cotton	Dermal	
*Clerodendrum myricoides* (Hochst.) R.Br. Ex Vatke	Verbenaceae			Leaf	Arthritis/rheumatism	Apply butter on patient’s head and let him/her fumigated with leaves of the plant	nasal	SA01347
					Conjunctivitis and trachoma	Leaves are crushed and added into boiling water and the patient steam baths himself	ophthalmic	
				Root	Evil eye	Roots are pounded into powder and sprinkled on fire to expel evil eye		
*Coffea arabica* L.	Rubiaceae	Buna	Shrub	Seed	Amoebiasis	Seeds are roasted, pounded into powder, mixed with honey and taken orally	oral	SA01397
					wound	Seeds are roasted, and pounded into powder and paste applied on affected part	Dermal	
					Fire burn	Seeds are roasted, pounded into powder and paste applied on affected area after mixing it with sap of *Aloe megalacantha*	Dermal	
*Colutea abyssinica* Jaub. and Spach.	Fabaceae	Qaqata	Shrub	Leaf	wound	Leaves are pounded into powder and sprayed on wound	Dermal	SA01342
*Commicarpus grandiflorus* (A. Rich.) Standl.	Nyctaginaceae	Ezni Tawa	Herb	Leaf	Furunclosis	Leaves are crushed and paste smeared on affected area	Dermal	SA01354
*Commiphora schimperi* (Berg) Engl.	Burseraceae	Anqa	Tree	Latex	wound	Latex smeared on wound	Dermal	SA01323
*Cordia africana* Lam.	Boragenaceae	Awhi	Tree	Leaf	febrile illness	Leaves are crushed, squeezed and liquid taken with coffee	oral	SA01367
*Croton macrostachyus* Del.	Euphorbiaceae	Tanbuk	Tree	Leaf	diarrhoea	Leaves are crushed squeezed and a cup of juice taken with honey	Oral	SA01373
				Bark	Bloat	A bark is dried, pounded into powder and one to two spoons of powder are added into coffee or tea and taken for a week	oral	
				Root	jaundice	Root bark is dried, pounded into powder and two to three spoons of powder are added into a cup containing water. Treatment is taken for 21 days	oral	
				Leaf, bark	Sudden stomach ache	Dried bark/leaves are pounded into powder, one to two tea spoon of powder are added into skimmed milk and served once	oral	
				Bark				
				Leaf	Malaria	Dried bark is pounded into powder, two to three spoons of powder added into local beer and taken for a week once per day. The medicine could cause diarrhoea and vomiting	oral	
					scabies	Leaves are crushed, mixed with butter and dressed on affected part	Dermal	
				Leaf sap	*Tinea versicolor*	Sap of leaves are applied on affected area	Dermal	
				Leaf				
					Urine retention	Leaves are added onto boiled water with sugar. Solution is then taken every morning for seven days	oral	
*Cucumis ficifolius* A. Rich.	Cucurbitaceae	Ramboramb, lomin bita	Herb	Leaf	Anthrax	Leaves are either ground into powder or crushed, squeezed, filtered, mixed with coffee and taken with a coffee cup for two days	oral	SA01321
				Root	Eye disease	Roots are chewed	oral	
					Jaundice	Roots are chewed	oral	
					Stomach ache	Roots are chewed	oral	
					Stomach ache following delivery	Roots are chewed	oral	
					Snake bite	Roots are chewed	oral	
				Fruit	Ear infection	Three drops of fruit juice are applied into ear for five days	ear	
					Tuberculosis	Roots are chewed	oral	
				Root	Teeth ache	Roots are chewed	oral	
				Fruit	Asthma	Fruits are washed, dried, ground into powder, added onto boiled coffee and drunk	oral	
				Root, leaf	Eczema	Roots and leaves are ground into powder, mixed with honey and dressed on affected area	Dermal	
				Fruit/leaf	Tetanus	Fruits and leaves are crushed, mixed with butter, heated on fire and applied on affected area and covered with clean cotton cloth. This is repeated for three days	dermal	
*Cucurbita pepo* L.	Cucurbitaceae	Duba	Herb	Seed	Tapeworm	Seven roasted seeds are taken orally, followed by three hours of fasting	oral	SA01390
				Fruit	Urine retention	Fruits are cooked and taken as soup	oral	
*Cynoglossum coeruleum* Hochst. ex A.DC.	Boraginaceae	Teng Begie	Herb	Leaf	Febrile illness (michi)	Leaves are crushed, squeezed and liquid taken with coffee or its lotion is applied on skin	Oral or dermal	SA01359
*Cyphostemma adenocaule* (steud.ex A. Rich) Descoings ex Wild and Drummond	vitaceae	Aserkuka fetahkuka	Climber	Root	Skull wound	Dried roots are ground into powder, mixed with butter and dressed on affected area	Dermal	SA01346
					Snake bite	Half of finger-sized root is chewed to detoxify poison	oral	
*Datura stramonium* L.	Solanaceae	mestenager	Herb	Leaf	Tetanus	Fresh leaves are crushed, mixed with butter, heated and smeared on affected area before covering it with clean cotton cloth	Dermal	SA01312
					Dandruff	Leaves are crushed and creamed on shaved head	Dermal	
				Seed	Teeth ache	Seeds are roasted on iron sheet and the patient inhales smoke	Oral/nasal	
					abortion	Half tea spoon of seeds are ground into powder, mixed with water and half of cup is drunk	oral	
				Leaf	Brain sharpness	Leaves are crushed, squeezed, filtered and a cup of juice is taken for some days	oral	
					Leishmaniasis	Leaves are crushed and pasted on affected area	dermal	
					Furunculosis	Leaves are crushed and pasted on affected area	Dermal	
					Herpes zoster	Leaves are roasted on iron sheet, pounded into powder, mixed with butter and smeared on affected area	Dermal	
					Scabies	Leaves are roasted on iron sheet, pounded into powder, mixed with butter and smeared on affected area	Dermal	
					eczema	Leaves are roasted on iron sheet and pounded in to powder. After mixed with pure butter smeared on affected area	Dermal	
*Dodonaea angustifolia* L. f.	Sapindaceae	Tahsos	Tree	Leaf	Herpes zoster	Leaves are roasted, ground into powder, mixed with butter and smeared on affected area	Dermal	SA01327
					wound	Leaf powder is sprayed on wound	Dermal	
*Erucastrum arabicum* Drummond and Hemsely	Brassicaceae	Hamli gudible	Herb	Leaf	Ring worm	Leaves are rubbed on skin	Dermal	SA01317
*Erythrina abyssinica* Lam. ex DC.	Fabaceae	Zuwabue, enqui hebey	Tree	Bark	Evil eye	Put bark on fire and let patient to fumigate himself with smoke	nasal	SA01322
*Eucalyptus globulus* Labill.	Myrtaceae	Tsada Kelamitose	Tree	Leaf	Febrile illness (michi)	The patient baths himself with steam of boiled leaves	Oral/nasal	SA01376
					*Tinea pedis*	Leaves are boiled in water and the patient washes his feet with the decoction	dermal	
*Euclea divinorum* Hiern.	Ebenaceae	Kuliew	Shrub	Root	Scorpion bite	Roots are chewed to relieve pain	oral	SA01379
				Root, stem	Rheumatism and arthritis	The patient spreads animal butter on his/her head, burn roots and stems on fire and baths him/herself with smoke	Dermal	
				Root	Urine retention	Roots are chewed	oral	
*Euphorbia cactus* Boiss	Euphorbiaceae	Kolqual hamat	Shrub	Latex	Leishmaniasis	Latex is smeared on affected area	Dermal	SA01386
					wound	Add few latex drops on wound	Dermal	
					Gonorrhoea and syphilis	Add three to four drops of latex on a piece of ‘enjera’ and eat it. Medicine is taken for five consecutive days. Overdose may cause diarrhoea and vomiting	oral	
				Root	Jaundice	Roots are ground into powder, mixed with honey and taken for seven days	oral	
				Latex	Ascariasis	Four drops of latex are mixed with sugar solution and taken once before diet	oral	
					leprosy	Latex smeared on affected area	Dermal	
*Euphorbia petitiana* A. Rich.	Euphorbiaceae	Demaito demu	Herb	Latex	Ring worm	Latex smeared on affected area	Dermal	SA01348
*Ficus palmata Forssk.*	Moraceae	Beless	Tree	Latex	Wart	Latex smeared on the first growing wart	Dermal	SA01304
					haemorrhoids	Latex smeared on affected area	Dermal	
					Wound	Dress wound with latex	Dermal	
*Foeniculum vulgare* Miller	Apiaceae	shelan	Herb	Whole plant	Urine retention	Take solution of the plant boiled in water	oral	SA01362
*Gomphocarpus fruticosus* (L.) Aiton f.	Asclepiadaceae	Demaito bereka	Herb	Latex	Ringworm	Dress latex on affected area	Dermal	
				Leaves, stem	Arthritis	Ground leaves and stems, mix powder with butter and apply on affected body. Patient needs to expose himself to sunlight for an hour	Dermal	SA01343
				Root	Abortion	Chew the root	oral	
*Gossypium herbaceum* L.	Malvaceae	Tut	Shrub	Root	Snake bite	Roots are chewed to detoxify poison	oral	SA01363
*Hibiscus micranthus* L.f	Malvaceae	Segot Hamat	Shrub	Whole plant	typhus	House is fumigated with smoke to protect oneself from the disease	nasal	
*Hypoestes forskaolii* (Vahl) R. Br.	Acanthaceae	Gerbia	Herb	Leaf	jaundice	Leaves are crushed, squeezed and juice taken orally		SA01315
*Jasminum granditlorum* L. *subsp. floribundum (*R.Br. ex Fresen.) P.S. Green	Oleaceae	habitselim	Shrub	Leaf	Ascariasis	Leaves are crushed, squeezed and cup of juice with sugar is taken orally	oral	SA01326
					tapeworm	Leaves are crushed, squeezed and cup of juice with sugar is taken orally	oral	
					wound	Leaves are roasted on iron sheet ground into powder and are sprayed on wound	Dermal	
					vomiting	Leaves are chewed to stop vomiting	oral	
*Justicia schimperiana* (Hochst. ex A.Nees) T. Anders	Acanthaceae	Shemeza	Shrub	Leaf	Jaundice	Seven leaves of *J. schimperiana* and seven leaves of *Croton mycrostachyus* roasted on iron sheet, crushed into powder are eaten with ‘enjera’ daily for twenty-one days	oral	SA01301
						A cup of leaf juice of the plant is taken daily for twenty-one days	oral	
*Klinia odora* Forssk.	Asteraceae	Berier	Shrub	Whole plant	Snake bite, evil eye, evil spirit	House is fumigated to repel snakes and expel evil spirit	nasal	SA01378
*Leonotis ocymifolia* (Bunn. f.) Iwarsson	Lamiaceae	Keyh Embeba Ketater	Herb	Whole plant	Febrile illness (michi)	Fumigating oneself with smoke of plant	nasal	SA01371
					Eye disease	Fumigating oneself with smoke of plant	nasal	
*Lepidium sativum* L.	Brassicaceae	Shenfa	Herb	Seed	Amoebiasis and diarrhoea	Seeds are ground into powder, mixed with honey and then taken for three days	Dermal	SA01310
					Gland TB	Open swelling/wound, add small amount of sulphur and covered it with seed paste of *L. sativum* and latex of *C. procera*	Dermal	
					Evil spirit	Grind seeds, add powder into water and spray solution indoor to expel evil sprit	Dermal	
					malaria	*L. sativum* seeds are crushed with leave*s* of *R. chalepensis* and *A. Sativum* and then taken orally for seven days	Dermal	
*Premna oligotricha* L.	Lamiaceae	Sasa hadima	Shrub	Leaf	Ascariasis	Leaves are crushed and squeezed and a cup of juice is taken once orally		SA01325
*Linum usitatissimum* L.	Linaceae	Entatie		Seed	Placental retention	Seeds roasted on iron sheet and grinding into powder, then cooked in the presence of honey and taken for a month before delivery	oral	SA01386
					amoebiasis	Seeds are ground, mixed with water and a cup of juice drunk in the morning	oral	
*Lycopersicon esculentum* Mill.	Solanaceae	Tsebhi Awun	Herb	Leaf	Anthrax	Leaves are crushed, mixed with honey and swallowed	oral	SA01352
*Maesa lanceolata* Forssk.	Myrsinaceae	Saira	Tree	Leaf	Scabies	Leaves are crushed and juice smeared on affected part	Dermal	SA01302
				Seed	Tapeworm	Seeds are ground, powder mixed with water and a cup of juice taken orally once	oral	
*Malva verticillata* L.	Malvaceae	Enkeftiha	Herb	Leaf	Anthrax	Leaves are crushed, mixed with honey and swallowed	oral	SA01330
*Melia azedarach* L.	Meliaceae	Neem	Tree	Leaf	Tonsillitis	Crush leaves, filter and drunk the juice	oral	SA01382
				Seed, leaf	Dandruff	Seeds and leaves are crushed and paste applied on head skin	Dermal	
				Leaf	Malaria	Leaves are crushed and squeezed, and a cup of solution taken orally daily for five days	oral	
					Tooth decay	Leaves are chewed and spat		
*Meriandra dianthera* (Roth, ex. Roem. & Schult.)Briq.	Lamiaceae	Mesaguh	Tree	Leaf	Blood pressure	Leaves are boiled in water and solution taken daily for a month by cup of tea until improvement	oral	SA01339
					Diarrhoea	Leaves are ground, powder is mixed with water and a cup of solution taken orally	oral	
					malaria	Leaves are crushed, squeezed and a cup of juice taken daily for five days		
*Nicotiana tabacum* L.	Solanaceae	Tunbako	Herb	Root	Snake bite	Roots are chewed or crushed and paste applied on wound	oral	SA01308
*Ocimum lamiifolium.* Hochst.Ex Benth.	Lamiaceae	Dem akher (demekasie)	Shrub	Leaf	Febrile illness (michi)	Leaves are crushed and solution drunk with coffee. Juice is also smeared on skin	oral/dermal	SA01311
*Olea europaea* L *s*ubsp. *cuspidata* (Wall. ex G. Don) Cif.	Oleaceae	Awlie	Tree	Leaf	Asthma	Leaves are boiled in water and a cup of solution drunk every evening with skimmed milk to arrest vomiting	oral	SA01374
					vomiting	Leaves are chewed to stop vomiting	oral	
					Amoebiasis	Leaves are crushed, squeezed and a cup of taken orally	oral	
					Eye infection	Leaves are crushed, squeezed, filtered and two to three drops are added daily into the eye for five days	ophthalmic	
					Teeth ache	Leaves are crushed and paste applied on affected area	Dermal	
					Ascariasis	Leaves are crushed, squeezed and a cup of juice taken orally for one day	oral	
*Ormocarpum pubescence* (Hochst.) Cuf. ex Gillett	Fabaceae	Alendia	Shrub	Stem	rheumatism	Stems burned on prepared place at home and females bathing the smoke putting butter on their head	nasal	SA01320
*Orobanche minor* Smit.	Orobanchaceae	Selmi	Herb	Whole plant	Eye disease	Burn the plant on clay dish and let the patient fumigate himself with smoke	nasal	SA01338
*Otostegia integrifolia* Benth.	Lamiaceae	Chendog	Shrub	Leaf	Blood pressure	Leaves are boiled boiling in water and a cup of solution drunk every morning until recovery	oral	SA01357
*Oxalis anthelmintica* A. Rich	Oxalidaceae	Habachego	Herb	Leaf	Heart failure	leaves are eaten for long period of time (about a year)	oral	SA01318
					Tapeworm	Patient eats some and remains on diet for next three hours	oral	
*Pavonia burchellii* (DC.) Dyer.	Malvaceae	Neger negarito	Shrub	Leaf	Stomach ache	Leaves are crushed, squeezed and a cup of juice taken orally		SA01388
					cough	A cup of leaf juice is taken orally		
*Phytolacca dodecandra* L’Herit.	Phytolacaceae	Shebti	Shrub	Root	Rabies	Dried root of the plant is powdered and mixed with local alcohol and a cup of solution drunk daily for twelve days. vomiting is its side effect and, therefore, restricted to children and pregnant women	oral	SA01387
				Leaf	Gonorrhoea	Leaves of *P. dodecandra* and roots of *C. macrostachyus* are ground, powdered mixed with water and solution drunk with one to two cups of coffee	oral	
					Jaundice	Leaves are crushed, squeezed and one cup of juice taken daily for 21 days	oral	
					scabies	Crushed leaves are rubbed on the skin. Skin is then washed in half –hour time	Dermal	
*Plantago lanceolata* L.	Plantaginaceae	Melhas kelbi	Herb	Leaf	*Tinea corperis*	Leaves are rubbed on affected area	Dermal	SA01358
					wound	Leaves are crushed, squeezed and solution applied on wound	Dermal	
*Plumbago zeylanica* L.	Plumbaginaceae	Aftihi	Shrub	Root	Evil eye, evil spirit, magic	Roots are fumigated in the house	nasal	SA01324
*Polygala abyssinica* Fres.	Polygalaceae	Etselebona	Herb	Root	Snake bite	Roots are chewed	oral	SA01314
					Sharpen mind	Finger-sized root is chewed. Overdose may causes madness	oral	
					Sever stomach ache	Roots are chewed	oral	
*Rhamnus prinoides* L’Herit.	Rhamnaceae	Gesho	Shrub	Leaf	Tonsillitis	Mothers chewing the leaves and spit to mouth of their children where as young ones chew it for themselves	Dermal	SA01350
					Eczema	Leaves crushed, mixed with pure butter and dressing the affected part	Dermal	
*Rhoicissus tridentata* (L. f.) Wild & Drummond	Vitaceae	Hareg temen (etsezewie)	Climber	Root	Snake bite	Less than a finger-sized root is chewed and swallowed. Overdose causes severe stomach ache and vomiting	oral	SA01344
*Rumex abyssinicus* Jacq.	Polygonaceae	mokemoko	Herb	Root	Blood pressure	Roots are ground, powder mixed with water and solution drunk with tea every morning until improvement	oral	SA01398
					Cancer	Root powder is mixed in spicy stew to increase its power of curing the disease	oral	
					Tooth ache	Chew root and apply paste on affected tooth	oral	
*Rumex nervosus* Vahl.	Polygonaceae	Huhot	Shrub	Stem	gastritis	Young stems are chewed with salt and swallowed	oral	SA01394
				Root	Snake bite	Roots are chewed to detoxify poison	oral	
				Leaf	Skin rash	Leaves are crushed and paste rubbed on affected area	Dermal	
					Breast cancer	Leaves are crushed and paste applied on affected area	Dermal	
*Ricinus communis* L.	Euphorbiaceae	Gulie	Shrub	Seed	Amoebiasis	Crushed seeds are mixed with water and taken with a cup of tea once	oral	SA01377
*Ruta chalepensis* L.	Rutaceae	Chena adam	Herb	Leaf	Evil eye	Rub the leaves and Smell	nasal	SA01380
					cough	Leaves boiled in milk are taken orally	oral	
					Malaria	Crushed the leaves of the plant with bulb of *A. sativum* in by adding and take medicine orally for three days	oral	
					Flue	Leaf of *R. chalepensis* is pounded with bulb of *A. Sativum*, mixed with soup and used as a drink	oral	
*Sansevieria erythraeae* Mattei	Dracenaceae	Eka termo	Shrub	Leaf	Ear infection	Leaves are heated on fire, juice squeezed into tea cup and three to four drops are added into the infected ear	inner	SA01365
*Schinus molle* L	Anacardiaceae	Tselim berbere	Tree	Stem	Blood pressure	Chewing the stem	oral	SA01364
				Leaf	Eye infection	Boil leaves in water and let the patient bath himself with steam	oral	
*Solanum hirtulum* Steud. ex A. Rich.	Solanaceae	Alalemo kelbi	Herb	Root	Stomach ache	Chewing the root	oral	SA01393
*Solanum incanum* L.	Solonaceaee	Neshtey engule	Shrub	Leaf	Anthrax	Seven leaves are crushed, mixed with honey and taken orally	oral	SA01372
				Root	Arthritis	Roots are ground, powder mixed with animal butter and cream applied on affected body part and let the patient expose himself to sun light for five days	Dermal	
					Stomach ache	Chewing the root	Dermal	
					Gonorrhoea	Roots are ground, powder mixed with honey and paste taken for five days	oral	
*Solanum marginatum* L. f.	Solanaceae	Abyiengule	Shrub	Seed	Tuberculosis	Seeds are dried, crushed and added into milk or coffee and solution taken every morning for 21 days	oral	SA01313
*Solanum nigrum* L.	Solanaceae	Alalemo Wezero	Shrub	Leaf	Epistaxis	Leaves crushed and pasted on the nasal openings	Dermal	SA01360
					Bleeding after delivery	Leaves are crushed and inserted into vagina	Dermal	
*Tagetes minuta* L.	Asteraceae	Etsefaruos	Herb	Whole plant	Evil eye	Smoking the plant and let the patient fumigate himself	nasal	SA01389
*Tragia uncinata* M. Gilbert	Euphorbiaceae	Amae	Herb	Root	Impotence	Roots are ground and taken orally with local soup for a week	oral	SA01361
*Trigonella foenum-graecum* L.	Fabaceae	Aba’ke	Herb	Seed	Urticaria	Grind seeds , mix powder with butter and apply cream on affected part	dermal	SA01392
					Stomach ache	Boil powder in water, add sugar and given to babies	oral	
*Verbascum sinaiticum* Benth.	Scrophulariaceae	Trnaka	Herb	Leaf	Bleeding	Leaves are crushed and paste applied on affected area	dermal	SA01366
					Haemorrhoids	Leaves are crushed, packed in a piece of cloth and inserted through rectum	rectal	
					Fire burn	Leaves are crushed, squeezed and juice applied on the damaged part using clean cotton	dermal	
					Swelling	Rub the swelling using fresh leaves	dermal	
*Verbena officinalis* subsp. *africana* R. Fernandes & Verdc.	Verbenaceae	Atush	Herb	Whole plant	Ascariasis	Plant is crushed, squeezed and juice taken with cup of coffee for three days		SA01307
					Diarrhoea	Plant is crushed, squeezed and juice taken with cup of coffee for two to three days	oral	
				Leaf	Ear infection	Leaves are crushed, squeezed, juice filtered with clean cotton cloth, juice mixed with goat butter and three drops are added into the infected ear	auricular	
					Herpes zoster	Leaves are crushed and paste applied on affected area	Dermal	
				Root	Snake bite	Chewing the root	oral	
					Tonsillitis	Adults chew the root and spit paste into the mouth of their sick child	oral	
				Whole plant	abdominal pain and febrile illness	Plant is crushed, squeezed and solution taken with the cup of tea	oral	
*Vernonia amygdalina* Del.	Asteraceae	Grawa	Tree	Leaf, root	Devil sickness	Rub body with crushed leaves or smoke root and inhale the smoke. Crushed young twigs and leaves may also be spread in a house	Dermal/nasal	SA01306
				Leaf	Malaria	Crushed leaves of this plant and *R. Chalepensis* are boiled and three tablet- sized medicine prepared by mixing paste with honey is served every morning for seven days	oral	
				Root	Snake bite	Chewing the root	oral	
				Leaf	Teeth ache	Leaves are chewed with bulbs of *A. sativum*	oral	
*Withania somnifera* (L.) Dunal	Solanaceae	Agoal	Shrub	Leaf, stem	michi	Leaves and stems of the plant are decocted with leaves of *E. globulus* and *C. africana* and patient takes steam nasally	nasal	SA01356
*Zehneria scabra* (Linn.f.) Sond.	Cucurbitaceae	Haregressa	Herb	Whole plant	Febrile illness	The plant together with *E. globulus* and *J. schimperiana* is boiled in water and patient takes steam nasally	Nasal	SA01305
*Zingiber officinale* Rosc.	Zingiberaceae	gengible	Herb	Rhizome	Blood pressure	Chewing the rhizome	Oral	SA01399
*Ziziphus spina-christi* (L.) Desf.	Rhamnaceae	geba	Shrub	Whole plant	Dandruff	Leaves are crushed and paste applied on head skin	Dermal	SA01370

**Table 2:** **List of medicinal plants used to treat livestock diseases**

**Scientific name**	**Family**	**Local name**	**Habit**	**Parts used**	**Disease treated**	**Animal treated**	**Mode of preparation and administration**	**Application route**	**Voucher no**
*Aloe megalacantha* Baker	Aloaceae	Ere	Shrub	Exudate	Anthrax	Cattle	Crush leaves, squeeze the exudate, mix it with cold water let the animal drink one cup of the solution	Oral	SA01384
					Trypanosomiasis	Cattle	Exudate is mixed with poultry faeces is smeared on affected body parts	Dermal	
				Root	Dislocation of body parts	Cattle	Roots are cut into pieces, tied by thread and tied on damaged part of the body	Dermal	
				Exudate	Wound	Cattle	Exudate is smeared on affected body part of the animal	Dermal	
						Equine			
						Sheep			
						Goat			
					Scabies	Cattle	Crush leaves and apply exudate on the infected skin	Dermal	
						Sheep			
						Goat			
*Achyranthes aspera* L.	Amaranthaceae	Muchelo	Herb	Root	Thelaziasis (eye disease)	Cattle	Roots are chewed and juice spitted into the affected eye of cattle	Eye	SA01303
						Equine			
*Allium sativum* L.	Alliaceae	Tsada shugurti	Herb	Bulb	Thelaziasis (eye disease)	Cattle	Bulbs are crushed, squeezed, filtered, mixed with soot and paste inserted into affected part	Eye	SA01368
						Equine			
					Aspergillosis	Cattle	Crush bulb with leaves of *Leucas* sp., squeezed it, add salt and administer a cup of the juice	Nasal	
						Equine			
						Sheep			
						Goat			
					Foot and mouth disease	Cattle	*Allium sativum* is crushed, mixed with honey and apply paste on affected part	Dermal	
					Newcastle disease	Poultry	Bulb is crushed, mixed with ‘enjera’ and is orally administered	Oral	
*Argemone mexicana* L.	Pappavaraceae	Medafe tilian	Herb	Leaf	Sore	Camel	Leaves are pounded into powder and sprayed on the wound daily after washing it with salted water	Dermal	SA01381
						Equine			
						Cattle			
*Calpurnia aurea* (Alt.) Benth.	Fabaceae	Hetsawets	Tree	Seed	Salmonellosis	Cattle	A cup of seeds are ground, powder mixed with salted cold water and solution given orally administered	Oral	SA01345
						Sheep			
						Goat			
				Leaf	*E. coli* infection	Cattle	Leaves are crushed, squeezed, filtered and juice is orally administered	Oral	
						Sheep			
						Goat			
					Lichen simplex chronicus (skin disease)	Cattle	Leaves are crushed and rubbed on the skin	Dermal	
					Sheep pox	Sheep	Leaves are crushed and rubbed on the skin	Dermal	
						Goat			
*Calotropis procera* (Ait.) Ait.	Asclepidaceae	Ginda	Shrub	Latex	Sore	Cattle	Latex smeared on affected area until cure	Dermal	SA01375
						Equine			
						Sheep			
						Goat			
*Croton macrostachyus* Del.	Euphorbiaceae	Tanbuk	Tree	Leaves	Scabies	Cattle	Leaf of *C. macrostachyus* is crushed and rubbed on the affected skin three to four days consecutively	Dermal	SA01373
						Goat			
						Sheep			
*Cucumis ficifolius* A. Rich.	Cucurbitaceae	rambo Rambo	Shrub	Root	infection	Equine	Roots is crushed into powder, mixed with cold water and a cup of solution is given orally	Oral	SA01321
						Cattle			
						Sheep			
						Goat			
					Hyena bite	Equine	Root is crushed, mixed with ‘tella’, decanted and paste applied on affected part	Dermal	
						Cattle			
*Cyphostemma adenocaule* (steud.ex A. Rich) Descoings ex Wild and Drummond	Vitaceae	Aserkuka fetahkuka	Climber	Root	Pack sore	Equine	Roots are crushed, dried, ground and powder sprinkled on affected part until sore dries	Dermal	SA01346
*Dodonaea angustifolia* L. f.	Sapindaceae	Tahsos	Shrub	Leaf	Sore on cattle and equine	Equine	Leaves are dried on hot iron plate, ground and powder spread on affected part	Dermal	SA01327
						Cattle			
						Sheep			
						Goat			
				Twig	Dislocation of body part	Cattle	Dislocated part is tied with twigs until healed	Dermal	
						Equine			
						Sheep			
						Goat			
*Eucalyptus globulus* Labill.	Myrtaceae	Tsada kelamitos	Tree	Leaf	Avian cholera	Poultry	Leaf of *E. globulus* is ground, powder boiled in water, solution added onto barely soup and fed to chicken	Oral	SA01376
*Euphorbia cactus* Boiss	Euphorbiaceae	Kolqual hamat	Shrub	Latex	Black leg	Cattle	Apply latex on the swollen part to protect the spread of the disease. Latex is also given in small amount with ‘enjera’	Dermal, oral	SA01386
*Justicia schimperiana* (Hochst. ex A.Nees) T. Anders	Acanthaceae	Shemeza	Shrub	Leaf, root	Blackleg	Cattle	Leaf and root of *J. schimperiana* is pounded with dried fruit of *Ricinus communis.* One bottle of the Solution is given to sick animal	Oral	SA01301
				Leaf					
					Parasites	Cattle	Pounded leaf of *J. schimperiana* is mixed with malt powder of barely and two to three glass of ‘tella’ given to the animal	Oral	
						Equine			
						Goat			
						Sheep			
*Lepidium sativum* L.	Brassicaceae	Shenfa	Herb	Seed	Dysentery	cattle	Seeds are crushed, powder mixed with finger milt bread and orally administered	Oral	SA01310
						sheep			
						goat			
					cenoresis	sheep	Crushed seed of *L. sativum* and bulb of *A. sativum* is are mixed with cold water and a cup of solution is given to the animal	Oral	
						goat			
					Diarrhoea	Cattle	Seed of *Lepidium sativum* are ground, powdered mixed with crushed bulb of *A. sativum* and given to the animal	Dermal	
					Bloating	Cattle	Seed of *L. sativum* and bulb of *Allium sativum* are crushed together, mixed with water and given to cattle	Dermal	
*Leucas abyssinica* (Benth.) Briq.	Lamiaceae	Sewa Kerni	Shrub	Leaf	Internal parasites	Sheep	Leaves are crushed and squeezed, mixed with crushed bulb of *Allium sativum*, solution is then filtered and applied nasally	Nasal	SA01383
						Goat			
*Premna oligotricha* L.	Lamiaceae	Sasa hadima	Shrub	Leaf	Internal parasites	Sheep	Leaves are crushed, squeezed and given to sick animal	Nasal	SA01325
						Goat			
				Leaf	Pasterellosis	Sheep	Leaves are pounded with bulb of *A. sativum*, squeezed and solution given to sick animal	Nasal	
						Goat			
*Linum usitatissimum* L.	Lineaaceae	Entatie	H.erb	Seed	Placental retention	Cattle	Seeds of *L. usitatissimum* are powdered and half a glass of powder is dissolved in water and given to cattle	Oral	SA01386
						Sheep			
						Goat			
*Melia azedarach* L.	Meliaceae	Nim	Tree	Leaf	Tick ulcer	Cattle	Leaves are rushed and rubbed on lymphagities ulcer	Dermal	SA01382
						Sheep			
						Goat			
*Nicotiana glauca* R. Grah.	Solanaceae	Tenbish/ cherged	Shrub	Leaf	External parasites	Cattle	Leaves crushed and rubbed on the skin of the animal	Dermal	SA01391
						Sheep			
						Goat			
*Nicotiana tabacum* L.	Solanaceae	Tumbako	Herb	Leaf, root	Plant toxin (toxicosis)	Cattle	Leaves and root are dried, powdered, mixed with salted water and a cup of the solution is given for one day the poisoned animal	Oral	SA01308
						Goat			
						Sheep			
				Leaf	Leech infestation	Cattle	Leaves are crushed, squeezed and a cup of solution is nasally applied	Nasal	
					Leech infestation	Cattle	Crushed and baked leaves are pounded, added on half litre of water and given to affected animal	Oral	
					Trypanosomiasis	Cattle	Leaves are crushed and baked, mixed with water and solution given to sick animal	Oral	
*Otostegia integrifolia* Benth.	Lamiaceae	Cheendog	Shrub	Whole plant	Ecto- parasites infestation	Cattle	Fumigate the plant in the house where the animals are kept	Dermal	SA01357
						Equine			
						Poultry			
						Goat			
						Sheep			
*Phytolacca dodecandra* L’Herit.	Phytolaccaceae	Shebti	Shrub	Leaf	Rabies	Cattle	Leaves are crushed with leaves of *C. mycrostachyus*, squeezed and a cup of juice mixed with ‘tella’ is given to the animal	Oral	SA01387
						Equine			
						Sheep			
						Goat			
					Scabies and external parasite infestation	Cattle	Leaves are crushed with little water and paste rubbed on the skin. The skin is then washed after thirty minutes	Dermal	
						sheep			
						Goat			
*Rhoicissus tridentata* (L. f.) Wild & Drummond	Vitaceae	Hareg temen	Climber	Root/Stem	Snake bite	Cattle	Root /stem is crushed, squeezed, mixed with cold water and a cup of solution is given only once to the animal	Oral	SA01344
						Goat			
						Sheep			
						Equine			
*Ricinus communis* L.	Euphobiaceae	Gulie	Shrub	Root	Sudden Sickness	Cattle	Roots of *R. communis* and *Justica schimperiana* are pounded, mixed with cold water and a cup of the solution is to the sick animal	Oral	SA01377
				Fruit	Anthrax	Cattle	Dried fruits are ground, powder mixed with cold water and a cup of solution is given the sick animal	Oral	
				Root	Actinomycosis	Cattle	Root is pounded by adding table salt and ash and mixed with water, solution is filtered and two glasses of it are to the sick animal	Oral	
						Sheep			
						Goat			
				Fruit	Epizoitic lymphagities	Cattle	Dried fruits are pounded and mixed with exudate of *Aloe megalacantha* and paste applied on ulcerated skin	Dermal	
						Equine			
						Sheep			
						Goat			
*Rhamnus prinoides* L'Herit.	Rhamnaceae	Gesh	Shrub	Leaf	Plant toxin (toxicosis)	Cattle	Leaves are crushed into powdered and mixed with malt of barely or oil or dissolved soap and one or two cups of the solution is given to the poisoned animal	Oral	SA01350
						Sheep			
						Goat			
*Ruta chalepensis* L.	Rutaceae	Chena adam	Herb	Leaf	Coccsidiosis	Poultry	Whole part of the plant, root of *J. schimperiana* and bark of *C. mycrostachyus* are pounded together and paste given to chicken by mixing it ‘enjera’ or water	Oral	SA01380
*Salvia schimperi* Benth.	Lamiaceae	Meshendedo	Herb	Leaf	Foot and mouth disease	Cattle	Leaves are crushed, mixed with honey and dressed on the affected part of the animal	Dermal	SA01355
						Goat			
						Sheep			
*Solanum marginatum* L. f.	Solonaceae	Abiyi engule	Shrub	Fruit	Urinary retention	Cattle	Fruits are crushed, pounded, two to three spoons of powder is mixed with cold water and a cup of solution is given to the sick animal	Oral	SA01313
						Sheep			
						Goat			
					Tuberculosis	Cattle	Fruits are crushed, pounded, two to three spoons of powder is mixed with cold water and a cup of solution is given to the sick animal until recovery	Oral	
						Sheep			
						Goat			

Relatively higher numbers of medicinal plants were used to treat intestinal parasites; diarrhoea and stomach ache (26 species), wounds, scabies and leprosy (23 species), respiratory disease (16 species), evil eye, evil spirit, devil sickness (15 species) and rheumatism and arthritis (15 species).

### Plant part (s) and methods used in preparation of remedies

**Figure 2:**
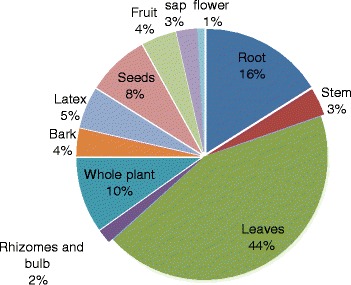
**Plant part(s) used in the study area for remedies preparations.**

**Figure 3:**
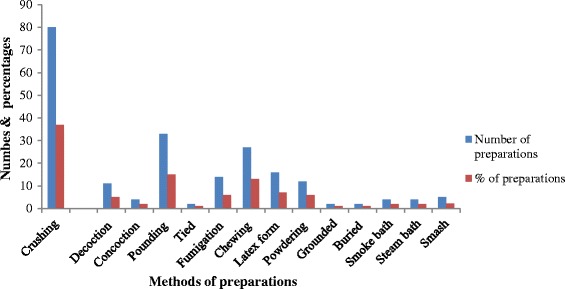
**Preparation methods of remedies.**

Leaves were the most preferred plants parts used in the preparation of remedies (44%), followed by roots (16%), whole plants (10%) and seeds (8%) (Figure 2). Crushing (37%), pounding (15%) and chewing (13%) were dominantly used in the preparation of remedies (Figure 3). Substances such as cold water, honey, coffee, butter, salt, sugar, soap, ash and milk were mixed with the plant materials during remedies preparations. The majority (60%) of remedies were prepared from fresh plant materials. Some (21.1%) were prepared from either dry or fresh materials and others (18.9%) from dry parts only.

### Route of remedy administration and dosage

Most medicinal plant preparations were applied internally (64.6%), out of which drinking took the lead (44.5%). Some are applied externally on the skin (35.4%), of which 42.5% are smeared on the skin (42.5%) (Table 3). Informants reported that dosages differed among traditional medicine practitioners even in treating the same health problem as remedies are prescribed with units of local measurement such as pinch, tea spoon (powder), tablet size of seed (semisolid), coffee cup, tea cup and water cup (liquid), finger length (root) and fist (leaves).

**Table 3:** **Route of administration of remedies**

**Main route of application**	**Mode of application**	**Percent applied**
**Internal application**		
	drinking	44.5
	chewing and swallowing	17
	swallowing	12
	Smoke bath	9.6
	nasal	6
	auricular	3
	ophthalmic	2
	Steam bath	2
	anal	1.4
	Buried	1.4
	**Total**	**100**
**External application**		
	Smearing	42.5
	pasting	20
	rubbing	12.5
	spraying	7.5
	Chewing and spitting	7.5
	washing	6
	tying	3.8
	**Total**	**100**

### Popularity of reported medicinal plants

*Cucumis ficifolius* is the most popular medicinal plant in the study area, cited by 81.8% of the informants, followed by *Allium sativum* (77%). Each of the medicinal plants *Croton marcostachyus, Ruta chalepensis* and *Vebena officinalis* were cited by 75.8% of the informants (Table 4).

**Table 4:** **Medicinal plants with highest informants’ consensus**

**Botanical name**	**Number (%) of informants who cited the plant**
*Cucumis ficifolius*	54 (81.8)
*Allium sativum*	51 (77)
*Croton macrostachyus*	50 (75.8)
*Ruta chalepensis*	50 (75.8)
*Vebena officinalis* subsp. *africana*	50 (75.8)
*Aloe megalocantha*	48 (72.7)
*Calotropis procera*	48 (72.7)
*Datura stramonium*	48 (72.7)
*Ocimum lamiifolium*	48 (72.7)
*Solanum incanum*	48 (72.7)
*Phytolacca dodecandra*	47 (71)
*Eucalyptus globulus*	46 (69.7)
*Olea europaea* subsp. *cuspidata*	46 (69.7)
*Plumbago zeylanica*	46 (69.7)
*Rhoicissus tridentata*	46 (69.7)
*Zehneria scabra*	46 (69.7)
*Cynoglossum coeruleum*	45 (68)
*Lepidium sativum*	45 (68)
*Withania somnifera*	45 (68)

### Informant consensus factor

Febrile illness is the disease group in the study area that scored the highest ICF value (0.97), followed by cardio-vascular problems (0.97), evil eye (0.95), hepatitis (0.95), warts and haemorrhoids (0.94), infectious wounds and scabies (0.92), snake and scorpion bites (0.92), fungal diseases (0.91) and intestinal parasites infection, diarrhoea and stomach ache (0.91) and malaria (0.91) (Table 5).

**Table 5:** **Informant consensus factor (ICF) values for aliments categories**

**Disease categories**	**No. of species**	**Species (%)**	**No. of use citations**	**Use citations (%)**	**ICF**
Abdominal irritation and vomiting	4	4.4	23	1	0.86
Bleeding and epistaxis	3	3.3	17	.8	0.88
Cardiovascular problems	5	5.6	85	3.7	0.95
Evil eye	15	16.7	287	12.6	0.95
Fungal diseases	14	15.6	138	6	.91
Head and tooth aches	8	8.9	60	2.6	0.88
Hepatitis	7	7.8	122	5	0.95
Infectious wounds and scabies	23	25.6	260	11	0.92
Intestinal parasites infection, diarrhoea and stomach ache	26	28.9	281	12	0.91
Malaria	8	8.9	79	3.5	0.91
Febrile illness	8	8.9	231	10	0.97
Non infectious swelling	10	11.1	87	3.7	0.89
Respiratory disease	16	17.8	136	6	0.89
Rheumatism and arthritis	15	16.7	105	4.6	0.87
Sensorial disease	11	12.2	102	4	0.90
Snake and scorpion bites	8	8.8	91	4	0.92
Urinary and placental retention	6	6.7	50	2.2	0.90
Venereal disease and reproductive organ problems	12	13.3	72	3	0.86
Warts and haemorrhoids	8	8.9	111	4.9	0.94

### Informants’ preference on medicinal plants used to treat snake bite

Preference ranking exercises of six selected informants indicate that *Rhoicissus tridentata* was the most preferred plant in treating snake bite, followed by *Nicotiana tabacum* (Table 6).

**Table 6:** **Preference ranking on selected plants used against snake bite**

**Medicinal plants**	**Respondents (A-H)**									
	**A**	**B**	**C**	**D**	**E**	**F**	**G**	**H**	**Total**	**Rank**
*Cucumis ficifolius*	3	2	1	4	3	2	3	5	23	6th
*Gossypium herbaceum*	3	2	4	5	4	2	3	4	27	5th
*Nicotiana tabacum*	5	3	4	5	5	3	3	4	32	2nd
*Rhoicissus tridentata*	6	5	3	6	5	5	4	3	37	1st
*Verbena officinalis*	6	3	2	4	3	3	2	5	28	4th
*Vernonia amygdalina*	5	4	4	3	5	2	4	3	30	3rd

### Multipurpose medicinal plants

The people in the study district relied on locally growing plant species for various purposes such as construction, firewood, medicine, charcoal, fencing, agricultural tool and furniture. Direct matrix ranking exercise performed on five commonly reported multipurpose medicinal plants shows that *Olea europaea* subsp. *cuspidata* was the most useful multipurpose plant, followed by *Cordia africana* (Table 7).

**Table 7:** **Results of direct matrix ranking on selected multipurpose medicinal plants**

	**Species**				
**Use category**	***Croton macrostachyus***	***Cordia africana***	***Maesa lanceolata***	***Olea europaea*** **subsp.** ***cuspidata***	***Acokanthera schimperi***
Firewood	2	3	3	4	3
Construction	3	4	4	4	3
Charcoal	2	2	3	4	2
Fencing	2	2	2	2	3
Agricultural instrument	4	3	3	4	1
Furniture	2	4	3	4	1
Medicine	4	3	4	4	4
Income source	2	4	2	4	2
Total	21	25	24	30	19
Rank	4th	2nd	3rd	1st	5th

### Habitats of and threats to medicinal plants

**Figure 4:**
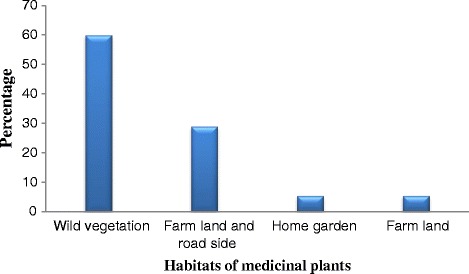
**Habitats from which medicinal plants were collected.**

The majority (60.2%) of medicinal plants were collected from the wild. Some (29%) were also collected from both farmlands and roadsides (Figure 4).

According to reports of informants, agricultural expansion is considered as number one threat to the survival of medicinal plants in the study area, followed by, cutting of trees for charcoal and fire wood consumption (Table 8).

**Table 8:** **Priority ranking of factors perceived as threats to medicinal plants**

**Factors**	**Respondents (R1-R7)**									
	**R1**	**R2**	**R3**	**R4**	**R5**	**R6**	**R7**	**Total**	**%**	**Rank**
Agricultural expansion	4	4	4	3	4	3	4	26	17.8	1st
Charcoal and fire wood consumption	4	3	4	4	3	3	3	24	16.4	2nd
Grazing	3	3	3	3	3	4	4	23	15.8	3rd
Settlement	2	2	3	2	2	3	2	16	11	6th
Timber and construction	2	3	4	3	3	1	4	20	13.7	4th
Drought	2	1	3	4	2	2	3	17	11.6	5th
Total								126		

Key: Values 1–4 were given: 1 is the least destructive threat and 4 is the most destructive threat.

### Marketed medicinal plants

Local market survey carried out in three towns of the District, namely Samre, Wenberta Adekeala and Fina Rewa revealed that plants were not sold in the markets for their sole medicinal purpose. The medicinal plants *Klinia odora*, *Lepidium sativum, Allium sativum, Rumex abyssinicus, Plumbago zeylanica, Linum usitatissimum and Ruta chalepensis* were sold primarily for their uses as species and food.

## Discussion

It is encouraging to find out that a high number of medicinal plants (90 species) are still being used by people in Seharti Samre District of Tigray Region, northern Ethiopia, to treat several human and livestock diseases. Ethnobotanical studies conducted in Ofla and Raya-Azebo districts of the same Region [6] came up with comparable numbers of medicinal plants, 83 and 60 species, respectively.

Several of the medicinal plants that were recorded from Seharti Samre District, were also mentioned in reports of studies previously conducted in Ethiopia, some of which (e.g. *Aloe* sp*, Ficus palmata, Justica schimperiana, Lepidium sativum, Linum usitatissimum, Nicotiana tabacum, Otostegia integrifolia, Ricinus communis, Rumex abyssinicus, Ruta chalepensis and Zehneria scabra*) [6,19] were exactly used for same medicinal purposes, which could be an indication of their pharmacological effectiveness.

Analysis of the data revealed Solanaceae, Lamiaceae and Fabaceae as the highest contributors of medicinal plants in the Seharti Samre District, which could be a reflection of their dominance in the flora of Ethiopia and Eritrea [20,21] in terms of their species richness. The study also showed that people in the study District use a relatively high number of shrubs and herbs, which is in agreement with studies conducted elsewhere in the country [22].

It was found out that two-third of medicinal plants in the study District were harvested from the wild, which is in agreement with reports of many studies conducted in the country [6,23,24]. Medicinal plants growing in the wild are highly exposed to different anthropogenic factors such as agricultural expansion, deforestation for charcoal and fire wood consumption, grazing, and harvesting for timber production and construction [22]. *Olea europaea* subsp. *cuspidata, Maesa lanceolata, Cordia africana*, *Croton mycrostachyus, Acokanthera schimperi, Phytolacca dedocandra*) are among the medicinal plants that were reported to be highly affected by the aforementioned factors.

Leaves and root were the most commonly used plant parts in the preparation of remedies in the study District. Many studies conducted in different parts of Ethiopia also showed that leaves are used more frequently than any other parts [6,25,26]. As compared to other parts, damage inflicted on medicinal plants due to harvest of leaves is very minimal [27].

Most of the medicinal plant species were reported to be processed through crushing followed by pounding and chewing. Ethnobotanical studies conducted in different parts of the country [9,25,26] reported similar results. Majority of the remedies in the study District were reported to be taken internally/orally followed by smearing on the skin. Several studies conducted in different parts of the county [28] also revealed that oral followed by dermal were the principal routes of remedy administration.

One of the major problems in traditional medicine is lack of standard dosages and précised measurements [5]. According to informants in the study District, the amount of dosage prescribed for same/similar health problems vary as remedies are prescribed with different units of local measurement. Inconsistency of doses has also been reported in studies conducted elsewhere in Ethiopia [6,29,30].

The study revealed that informants above the age of 40 years had relatively better knowledge of medicinal plants as compared to the younger ones (20 to 40 years old). Similar study conducted among the Zay community in Ethiopia [24] revealed that 90% of the elders above 40 years of age had rich medicinal plant knowledge. Study conducted in Nigeria [31] reported that the highest percentage of younger generation had no any knowledge of traditional medicine practice due to more exposure to modern life style. This may demonstrate the impact of modernization on medicinal plant use and transfer of the associated knowledge to the younger generation. The fact that most of the knowledge on traditional medication is kept with elders for the sake of secrecy, gaining respect and generating income is believed to contribute towards depletion of the same as generation passes by.

## Conclusion

A total of 90 medicinal plants were reported by informants from the study District. As most of the medicinal plants were harvested from the wild, appropriate conservation measures are required to ensure their sustainable harvesting besides to efforts of aawareness creation among the community by concerned bodies regarding the usefulness of their medical plants. The efficacy and safety of the claimed medicinal plants need to be evaluated before recommending them for their wider use. Priority should be given to medicinal plants with the highest informant agreement as such plants are believed to have better activity.
